# The association between nap time, nighttime sleep and depression in Chinese older adults: A cross-sectional study

**DOI:** 10.1371/journal.pone.0302939

**Published:** 2024-06-06

**Authors:** Yanliqing Song, Haoqiang Liu, Yue Liu

**Affiliations:** 1 College of Sports, Nanjing Tech University, Nanjing, China; 2 School of Athletic Performance, Shanghai University of Sport, Shanghai, China; Federal University of Rio Grande do Sul: Universidade Federal do Rio Grande do Sul, BRAZIL

## Abstract

**Objective:**

To explore the relationship among nap time, night sleep time, and depression among the elderly and to determine the recommended sleep time to provide a scientific and reasonable basis for the prevention and control of depression in residents.

**Methods:**

Based on the 2020 China Health and Elderly Care Longitudinal Survey (CHARLS) database, the demographic data and the health and lifestyle information of the study subjects were obtained. A total of 2,959 valid samples were included, and the relationship between sleep and depression was explored by logistic regression, restricted cubic spline, and isotemporal substitution model.

**Results:**

In the cross-sectional analysis, no statistical relationship was observed between napping time and depression in the elderly. The optimal sleep interval for the elderly at night is 6–7.5 hours, and the health benefits are the largest. A sleep duration of < 6 hours at night (OR = 2.25, 95% CI: 1.90 to 2.65) was associated with a high likelihood of depression. The probability of depression in the elderly continues to decrease with the increase of time after the nighttime sleep duration reaches 6 hours and is at the lowest level of about 7.5 hours. Moreover, the probability of depression will increase after the sleep duration exceeds 9.5 hours. In the range of 6–7.5 hours of recommended sleep duration, the likelihood of depression in the elderly will be reduced by 0.311 for every 30-minute increase in nighttime sleep time instead of noon sleep time.

**Conclusion:**

The duration of nighttime sleep and the probability of depression have a U-shaped relationship. The likelihood of depression was lowest in the elderly who slept for 6–8 hours at night, and the likelihood of depression could be reduced by increasing the nighttime sleep time instead of napping time within the optimal nighttime sleep range.

## Introduction

Depression is a serious public health problem worldwide [1]. According to the World Health Organization, more than 1 billion people are currently over the age of 60 [2]. As the elderly age, the body ages as well. Along with decreased levels of health, the death of partners and friends, a narrowing of social circles, and a decline in the level of social support, aging often leads to low self-esteem and further depression in older people [3, 4]. Depression affects about 300 million people worldwide and is one of the most common mental disorders in the general population and one of the top three causes of disability [5, 6]. According to the China Mental Health Survey, only 0.5% of people with depression in China received adequate treatment in 2021 [7]. In addition, the total cost of health care is 47%–51% higher for older adults with depression than for those without depression [8].

Sleep is another important public health topic. Previous studies have shown that sleep duration has been linked to numerous health problems [9, 10]. Sleep has been associated with depression as a modifiable lifestyle factor in many studies [11, 12]. For instance, researchers have reported a complex correlation between depression and changes in nighttime sleep duration in Chinese adults from 2011 to 2013 [13]. In people younger than 65 years of age, a positive association was observed between short periods of sleep during the day and depression [14]. Some studies have also specifically explored the relationship between sleep quality and depression among older Chinese people [15, 16]. However, no studies have focused on the relationship between midday sleep time, nighttime sleep time, and depression in older adults. Notably, 22%–69% of the world’s older adults have a napping habit during the day [17]. Especially in China, napping after lunch is popular because it is culturally considered a healthy lifestyle habit [18, 19]. A cross-sectional association between prolonged daytime napping and cognitive decline has been reported in older Chinese adults [20]. However, a cross-sectional association between daytime napping and depression remains unclear.

The purpose of this study was to explore the relationship among nap time, night sleep time, and depression in the elderly and to determine the recommended sleep time. This examination seeks to provide a scientific and reasonable basis for the prevention and control of depression in residents.

## Methods

### Study population

The data for this study were derived from the 2020 China Health and Retirement Longitudinal Study (CHARLS). CHARLS refers to the first nationally representative survey of middle-aged and elderly population in China, and it adopts a multistage PPS random sampling method based on implicit stratification to ensure a well-represented sample [21]. The survey was completed between April and October 2020.The CHARLS questionnaire provides data on sociodemographics, psychological status, health status, and other modules and covers tens of thousands of research subjects in 28 provinces, autonomous regions, and municipalities directly under the central government, which represent 95% of China Population. The 2020 CHARLS survey was approved by the Biomedical Ethics Committee of Peking University under the approval number IRB00001052-11015. During the field survey, each respondent who consented to participate in the survey was asked to sign two informed consent forms. One copy is kept by the interviewee himself, and the other is deposited in the CHARLS office. The inclusion criterion was older adults aged 60 years and older. Missing samples of demographic variables and missing samples of Epidemiological Center Depression Scale scores were excluded. A sample consisting of 7208 cases was finally included in this study.

### Measures

#### Outcome

Night sleep duration was assessed based on the following question: “How many hours did you actually fall asleep each night in the past month? (the average time in a night, probably shorter than the amount of time you spent in bed),” which represents the average period of a typical workday and rest day. Previous studies on sleep duration revealed the benefits of 6–8 h of sleep [22], and a meta-analysis of 35 sleep studies divided the study participants into three types of sleep duration: short (<6 h), moderate (6–8 h; reference group), and long sleep (>8 h) [23]. Nap time was assessed based on the following question: “How long did you usually nap in the past month?” The participants were divided into four groups: no-nap (0 min), short-nap (<30 min), moderate-nap (30–90 min), and long-nap (>90 min) groups. These cut-points were selected with reference to epidemiological literature on daytime napping [24].

#### Explanatory variable

The Chinese version of The Center for Epidemiologic Study depression (CES-D) was used to identify possible depressive symptoms. Cronbach’s α, with a value of 0.815, was used to verify the reliability and validity of the study [25]. Boey (1999) reports a validation exercise of the 10 question CES-D among elderly respondents in China. The Chronbach’s a of 0.78–0.79, which indicates a reasonable level of internal consistency [26].Based on the overall cutoff score recommended by the original compiler, Radloff, the 80th percentile (17 points) of the participants’ depression scale score distribution, was used as cutoff point for depressive symptoms [27].

#### Other covariates

For sociodemographic covariates, we selected variables, including gender (female or male), region (urban, urban-rural junction, or rural), marital status (married, divorced, widowed, or unmarried), health self-rating (very bad, bad, fair, good, or very good), education level (illiterate, primary, or junior high school and above), chronic disease (yes or), smoking (yes or no), alcohol consumption (yes or no), physical activity level (low intensity or moderate to high intensity).

Physical activity level: The CHARLS questionnaire divides the physical activity of the elderly into three levels: high, moderate, and low intensities, including the number and duration of physical activity of different intensities per week [28]. More than 3 h of physical activity were recorded per day at different intensities to “180 minutes.” Only the physical activity of different intensity types were allowed and quantified at a maximum of 21 h per week to avoid misclassification of the samples into the high-intensity physical-activity group Based on the assigned metabolic equivalents (METs), different types of physical activity were observed at various intensity levels in the Summary of Physical Activity (8.0 for high-intensity physical activity, 4.0 for moderate-intensity physical activity, and 3.3 for low-intensity physical activity) [29]. One week of physical activity energy expenditure was obtained using the formula (1 week of physical activity energy expenditure = metabolic equivalent of physical activity corresponding to the type of intensity *number of days of physical activity corresponding to intensity per week* time) [30]. In accordance with the International Physical Activity Scale, the physical activity of the elderly was divided into three levels: high (>3000 METs/week), moderate (600–3000 METs/week), and low (<600 METs/week) intensities [31].

#### Statistical analysis

Statistical analysis was performed using the R 4.2 software. The baseline characteristics of different types of sleep duration in older adults were described using the mean and standard deviation (M±SD) of continuous variables and the frequency and percentage (N, %) of categorical variables. Univariate analysis was performed to test for continuous variables with variance (ANOVA) and categorical variables with χ2, and the nap and nighttime sleep durations were used as reference groups to construct a logistic regression model. The multicollinearity between independent variables is measured by the variance inflation factor(VIF), The Box-Tidwell method was used to test the linear relationship between logarithmic values of independent and dependent variables in continuous type.Model 1 includes nighttime and nap time of the elderly, model 2 controls for demographic variables, and model 3 has all covariates. A restricted cubic spline plot was drawn to explore the dose–response relationship between sleep duration and depression in the elderly. In addition, compositional isotemporal substitution analysis was conducted to evaluate the cross-sectional association among nighttime sleep duration, noon sleep duration, and depression in older adults. Before running the model, the time for all sleep types (nighttime sleep, noon sleep, total sleep time) was divided by a constant of 30 as the time unit for 30 min, with each additional unit representing a 30 min increase per day. The nighttime sleep time was added to noon sleep time, and a variable representing the total sleep duration for isochronous substitution was established. The P values were used in a two-sided test, with P < 0.05 considered statistically significant.

## Results

### Participant characteristics

The effective sample comprised 2959 elderly people, including 1391 females (47.01%). The average age of the elderly was (67.50±5.83) years, and 1273 (43.1%) had depressive symptoms. Statistically significance was observed in the differences in gender, education level, health self-evaluation, chronic diseases, smoking, depressive symptoms, night sleep time, alcohol consumption, and noon sleep time(P<0.05), and the specific basic characteristics are shown in Table 1.

**Table 1:** Basic characteristics of study participants(n = 2959).

Variable	ALL (n = 2959)	Normal group (n = 1686)	Depression group (n = 1273)	χ^2^	P
age group, n (%)				χ^2^ = 0.976	0.614
60–69 years old	2080 (70.29)	1173 (69.57)	907 (71.25)		
70–79 years old	783 (26.46)	457 (27.11)	326 (25.61)		
80 years of age and above	96 (3.24)	56 (3.32)	40 (3.14)		
region, n (%)				χ^2^ = 3.580	0.167
city	500 (16.91)	293 (17.39)	207 (16.27)		
Urban-rural junction	280 (9.47)	172 (10.21)	108 (8.49)		
countryside	2177 (73.62)	1220 (72.40)	957 (75.24)		
gender, n (%)				χ^2^ = 97.056	< .001
female	1391 (47.01)	925 (54.86)	466 (36.61)		
male	1568 (52.99)	761 (45.14)	807 (63.39)		
Level of education, n (%)				χ^2^ = 51.292	< .001
illiterate	892 (30.15)	437 (25.92)	455 (35.74)		
elementary school	1793 (60.59)	1051 (62.34)	742 (58.29)		
Junior high school and above	274 (9.26)	198 (11.74)	76 (5.97)		
Marital status, n (%)				χ^2^ = 0.383	0.944
married	2274 (76.85)	1295 (76.81)	979 (76.90)		
divorce	24 (0.81)	15 (0.89)	9 (0.71)		
widowed	641 (21.66)	364 (21.59)	277 (21.76)		
unmarried	20 (0.68)	12 (0.71)	8 (0.63)		
Health self-assessment, n (%)				χ^2^ = 261.843	< .001
very bad	174 (5.88)	47 (2.79)	127 (9.98)		
bad	547 (18.49)	205 (12.16)	342 (26.87)		
fair	1611 (54.44)	946 (56.11)	665 (52.24)		
good	348 (11.76)	270 (16.01)	78 (6.13)		
very good	279 (9.43)	218 (12.93)	61 (4.79)		
Suffering from a chronic disease, n (%)				χ^2^ = 17.836	< .001
No	340 (11.49)	230 (13.64)	110 (8.64)		
yes	2619 (88.51)	1456 (86.36)	1163 (91.36)		
Intensity of physical activity, n (%)				χ^2^ = 3.723	0.054
LPA	45 (1.52)	32 (1.90)	13 (1.02)		
MVPA	2914 (98.48)	1654 (98.10)	1260 (98.98)		
smoking n (%)				χ^2^ = 36.659	< .001
No	1824 (61.64)	960 (56.94)	864 (67.87)		
yes	1135 (38.36)	726 (43.06)	409 (32.13)		
Depressive symptoms, n (%)				χ^2^ = 2959.000	< .001
No	1686 (56.98)	1686 (100.00)	0 (0.00)		
yes	1273 (43.02)	0 (0.00)	1273 (100.00)		
Nighttime sleep, n (%)				χ^2^ = 167.097	< .001
< 6h	1232 (41.64)	532 (31.55)	700 (54.99)		
6–8 h	1502 (50.76)	1016 (60.26)	486 (38.18)		
>8 h	225 (7.6)	138 (8.19)	87 (6.83)		
drink, n (%)				χ^2^ = 27.341	< .001
No	1881 (63.57)	1004 (59.55)	877 (68.89)		
yes	1078 (36.43)	682 (40.45)	396 (31.11)		
Nap sleep time, n (%)				χ^2^ = 9.455	0.024
0 min	1059 (35.79)	570 (33.81)	489 (38.41)		
<30 min	481 (16.26)	267 (15.84)	214 (16.81)		
30–90 min	818 (27.64)	488 (28.94)	330 (25.92)		
>90 min	601 (20.31)	361 (21.41)	240 (18.85)		

Note: LPA: Low intensity physical activity, MVPA: *Moderate to vigorous physical activity*.

### Logistic regression of the relationship between sleep duration and depression in older adults

For multicollinearity diagnosis, VIF values greater than 10 are considered to be multicollinearity and will be eliminated. The VIF values of our data here are all below 10, and there are no variables that need to be eliminated. The Box-Tidwell test indicated that the relationship between the continuous independent variables (age, intensity of physical activity, nighttime sleep, nap sleep time) and the dependent variable (depression) was linear (P >0.05). Logistic regression model analysis showed that after controlling for all covariates in model 3, 6–8 and more than 8 h of nighttime sleep were protective factors for the likelihood of depression compared with the elderly who slept <6 h at night. Any length of napping in older adults is not an influencing factor for the likelihood of developing depression(Table 2).

**Table 2:** Logistic regression table of the relationship between sleep duration and depression in the elderly.

Variable	Model one	Model two	Model three
gender			
female		1.00 (Reference)	1.00 (Reference)
male		1.78 (1.51–2.11)**	1.89 (1.48–2.42)**
Level of educatio			
illiterate		1.00 (Reference)	1.00 (Reference)
elementary school		0.88 (0.73–1.05)	0.87 (0.72–1.04)
Junior high school and above		0.54 (0.40–0.74) **	0.58 (0.42–0.80) **
Health self-assessment			
very bad			1.00 (Reference)
bad			0.62 (0.42–0.92) *
fair			0.29 (0.20–0.42) **
good			0.13 (0.08–0.20) **
very good			0.12 (0.08–0.19) **
Suffering from a chronic disease			
No			1.00 (Reference)
yes			1.02 (0.78–1.32)
smoking			
No			1.00 (Reference)
yes			1.12 (0.88–1.42)
drink			
No			1.00 (Reference)
yes			0.97 (0.81–1.16)
Nighttime sleep (h)			
< 6h	1.00 (Reference)	1.00 (Reference)	1.00 (Reference)
6–8 h	0.37 (0.31–0.43)**	0.39 (0.34–0.46) **	0.45 (0.38–0.53) **
>8 h	0.49 (0.36–0.65)**	0.49 (0.36–0.66) **	0.55 (0.40–0.75) **
Nap time (min)			
0 min	1.00 (Reference)	1.00 (Reference)	1.00 (Reference)
<30 min	0.96 (0.77–1.21)	1.02 (0.81–1.28)	1.04 (0.82–1.32)
30–90 min	0.89 (0.74–1.08)	0.98 (0.80–1.19)	1.01 (0.82–1.23)
>90 min	0.86 (0.70–1.07)	0.97 (0.78–1.20)	0.99 (0.80–1.24)

Note: **Description P<0.01, *Description P<0.05; model 1 includes nighttime and nap time of the elderly, model 2 controls for demographic variables, and model 3 has all covariates.

### Dose–response relationship between nighttime sleep duration and depression in older adults

**Fig 1:**
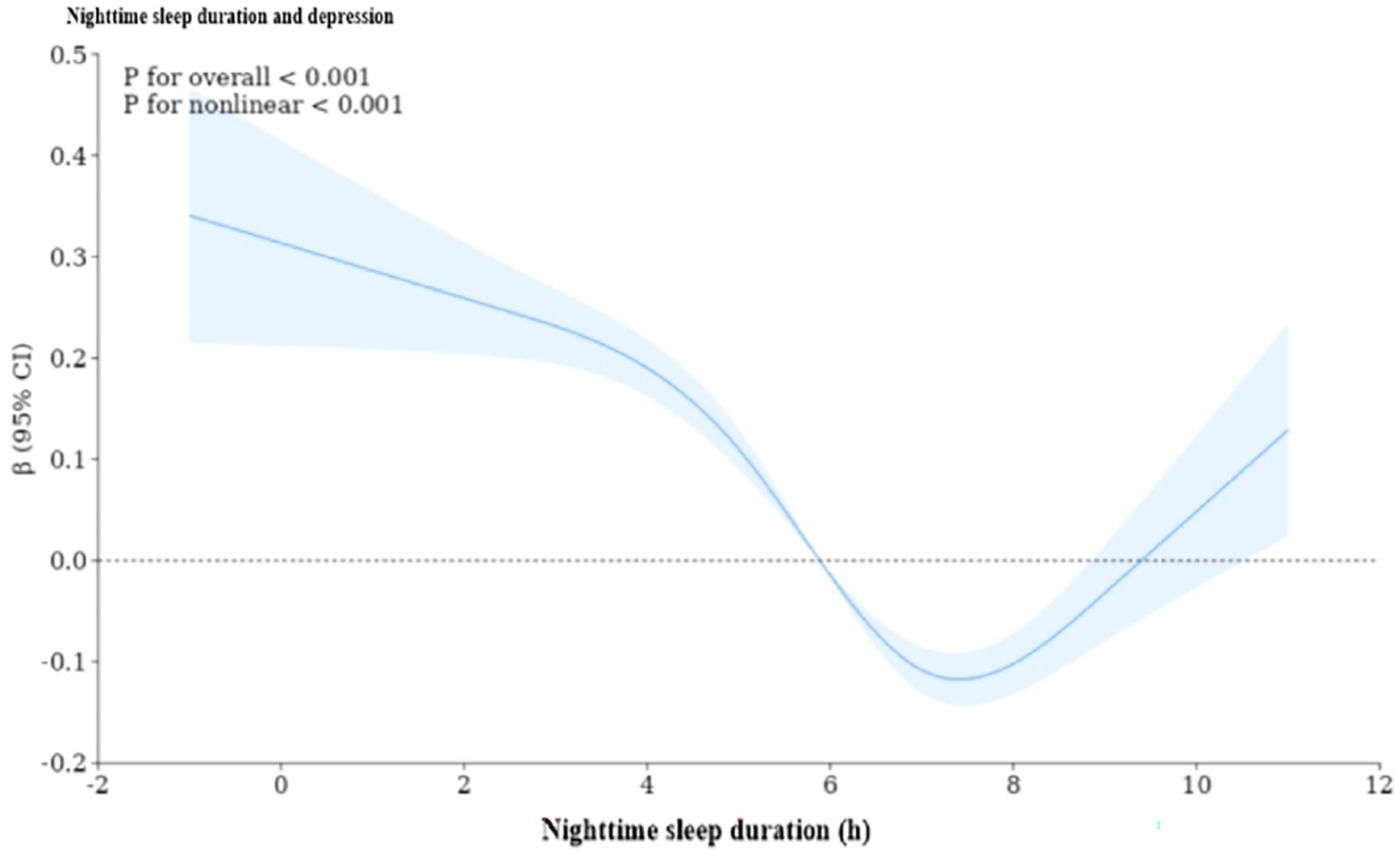
Dose-response relationship between nighttime sleep duration and depression in the elderly. The x-axis represents nighttime sleep duration in hours. The y-axis indicates the β-value calculated by the model. *The* shadowed area represents *the 95*% *confidence interval* (test for overall trend: *P* < *0*.*001;* test for nonlinear trend: *P* < *0*.*001)*.

The dose–response relationship between nighttime sleep duration and depression in the elderly showed that the former was U-shaped (P-nonlinear<0.001), and the likelihood of depression in the elderly decreased from 6 h of sleep to approximately 7.5 h. In addition, the probability of depression increased after more than 9.5 h of sleep (Fig 1).

Given that the probability of depression in the elderly was at the lowest level of 6–8 hours of sleep at night, the logistic regression model was constructed with 6–8 hours as the control group (Table 3) compared with the 6 h of night sleep < in the elderly. The 6–8 h of night sleep was a protective factor for the occurrence of depression.

**Table 3:** Logistic regression to adjust the relationship between nighttime sleep grouping and depression in the elderly.

Variable	Beta	S.E	Z	P	OR (95%CI)
Nighttime sleep time(h)					
6-8h					1.00 (Reference)
< 6 h	0.81	0.08	9.59	< .001	2.25 (1.90–2.65)
>8 h	0.20	0.16	1.30	0.195	1.22 (0.90–1.66)

Note: The model adjusted for gender, education level, and health self-assessment.

### Compositional isotemporal substitution relationship between nighttime sleep and nap time and depression in the elderly

Table 4 shows that after controlling for gender, education level, and health self-rating variables, 30 min of nighttime sleep was associated with the occurrence of depression in the elderly (β = -0.311, 95% CI: -0.469, -0.153, P<0.01), which indicates that their depression score was reduced by 0.311 for every 30 min increase in nighttime sleep instead of nap time,

**Table 4:** A compositional isotemporal substitution analysis table.

Variable	Beta	SE	t	p	95% CI
constant	16.241	0.713	22.785	0.000	(14.843–17.639)
gender	1.583	0.234	6.690	0.000	(1.105–2.021)
Educational attainment	-0.989	0.195	-5.077	0.000	(-1.371- -0.607
Health self-assessment	-1.968	0.114	-17.249	0.000	(-2.191- -1.744)
Nighttime sleep time	-0.311	0.080	-3.868	0.000	(-0.469- - 0.153)
Total sleep time	0.003	0.072	0.039	0.969	(-0.139–0.145)

Note: The regression coefficient is the effect of 30 min nighttime sleep instead of 30 min nap time.

## Discussion

### Major findings

In the cross-sectional analysis, the optimal sleep interval for the elderly at night was 6–7.5 hours, and the health benefit was the largest during this period. A sleep duration of < 6 hours at night (OR = 2.25, 95% CI: 1.90 to 2.65) was associated with a high likelihood of depression. The duration of nighttime sleep had a U-shaped relationship with the likelihood of depression. As such, the probability of depression in the elderly will continue to decrease with the increase of time after the nighttime sleep duration reaches 6 hours and is at the lowest level of about 7.5 hours. However, the likelihood of depression will increase after the sleep duration exceeds 9.5 hours. In the range of 6–7.5 hours of sleep duration, the depression score of the elderly decreased by 0.311 for every 30-minute increase in nighttime sleep time instead of noon sleep time. No statistically significant relationship was observed between nap length and likelihood of depression in older adults.

### Comparison with previous study

Previous studies have not specifically explored the association between depression and nap time in older adults. Few studies have focused on the relationship between daytime napping and depression. A trend analysis of 500,000 Chinese adults from 10 regions showed a positive correlation between daytime napping and depression, and this relationship was only significant in people younger than 65 years old [14]. In studies on older adults, few researchers have reported an association between daytime napping and depression [32–34].

Studies have shown that increasing the amount of nightly sleep reduces the likelihood of depression. While a long amount of sleep does not necessarily mean better sleep quality, a few more hours of sleep can compensate for one’s lack of sleep, which can improve feelings of fatigue and promote physiological recovery [35]. However, increasing one’s sleep time does not necessarily entail that long hours of sleep are always beneficial. Prolonged sleep may lead to oversleeping, which can have detrimental effects physically and psychologically.

This study further explores the extent to which incremental changes in sleep duration are beneficial. Notably, the duration of nighttime sleep has a U-shaped relationship with the likelihood of depression. Previous studies have also shown a U-shaped relationship between sleep time and depression in adolescents and adults [36, 37]. This study found no association between sleep duration of more than 8 hours and depression. Moreover, older adults who slept less than 6 hours were at increased likelihood of depression. This outcome is consistent with the results of a 2011–2013 Chinese study, which found no significant association between depression and long sleep duration among Chinese older adults and a significant association between depression and sleep deprivation [13]. A significant cross-sectional correlation between excessive sleep time and depression in older adults has also been reported [38–40]. The results may have been inconsistent because the statistical results are not significant because of the small sample size of older adults who sleep for long periods [41]. This study found no difference in the likelihood of depressive between long sleep duration (> 8 hours/day) and normal sleep duration (6–8 hours/day), which is consistent with the conclusions of previous studies [42]. Another study also found a U-shaped relationship between sleep time and depression in older adults, with an 86% increased probability of sudden depression during short sleep and a 49% increase in prolonged sleep compared with normal sleep time. The researchers further used a two piecewise linear regression model and found that compared with older adults who slept for < 8 hours, the likelihood of sudden depression decreased by 32% in older adults who slept adequately for 8 hours. Compared with those who slept adequately for 8 hours, the probability of sudden depression increased by 32% in those who slept > 8 hours [43]. In this study, we found that the incremental change of sleep time was 6–8 hours, and the inflection point was 7.5 hours.

Daily behaviors are interdependent, and the time used to allocate sleep comprises nighttime sleep and midday sleep [44, 45]. Therefore, the increase in the time spent on one type of sleep behavior, such as nighttime sleep, must be compensated by a decrease in other types of behaviors, such as midday sleep. Our study was the first to use time-use epidemiology to examine the relationship between all-day sleep and health outcomes [46]. Studies have reported harmful effects of daytime napping, including the disruption of circadian rhythms and altered serum levels, both of which have also been linked to depression [47, 48]. This study suggests that nap time can be replaced by increasing the time of sleep at night. In a reasonable sleep period of 6–7.5 hours, the likelihood of depression will be reduced for every 30 minutes of sleep time in the evening instead of midday sleep time in the elderly.

### Limitations

Our study has certain limitations. First, these results are based on cross-sectional data and do not allow us to determine the causal direction of the observed associations. Second, given that the CHARLS survey was not specifically conducted in the elderly and the survey period was during the 2020 COVID-19 pandemic, survey information was difficult to obtain. As a result of the lack of key data, the study sample missing rate was more than 30%, which caused a certain bias to the research results. The determination of depressive symptoms was based on standardized questionnaires rather than clinical psychiatric examinations, thus undermining confidence in the results. At the same time, in the “Other covariates” section. We selected several sociodemographic variables, but none of them related to whether the subjects were working or retired. If some of them were still working, this variable could interfere with the results. Since the survey subjects of this study are the elderly in China, 99.7% of the elderly have not gone through the retirement procedures, according to the questionnaire survey data, and after referring to some previous literature [49, 50], this variable was not included, and this influencing factor will definitely be considered in further research in the future to reduce the bias of the research results.However, we only used the CES-D to assess depressive symptoms, which is not the gold standard method for diagnosing depression and may over- or underestimate depressive symptoms. However, the CESD-10 tool has been validated in the Chinese population and has shown good validity, which also improves the reliability and validity of the results. Finally, given the lack of valid scales, the information collected only includes self-reported sleep duration, sleep quality, and whether sleep disorders also affect depressive symptoms. Data on sleep status and sleep quality are also lacking. Hence, analyzing the quantitative association between sleep and depression further becomes impossible.

## Conclusions

Our findings suggest that no association exists between midday sleep duration and depression after controlling for potential confounders. The duration of nighttime sleep has a U-shaped relationship with the likelihood of depression. As such, the likelihood of depression in older adults can be reduced by increasing the number of nighttime sleeps instead of nap times within the optimal nighttime sleep range.

## Supporting information

S1 Dataset (XLSX)
